# Supplementary material for: Evaluating the effect of a community score card among pregnant and breastfeeding women living with HIV in two districts in Malawi
Source: PLoS One. 2021 Aug 11;16(8):e0255788. doi: 10.1371/journal.pone.0255788 (PMC8357126; doi:10.1371/journal.pone.0255788)
Supplement: Attachment — Submitted filename: Response to reviewers 3.31.21.docx [file pone.0255788.s009.docx]

| **Reviewer** | **Comment** | **Revision made** |
| --- | --- | --- |
| 1 | *Not clear why the study took place in only the specified targeted districts since the data used to express the gap had been national level data e.g national level prevalence, retention rate, and EID uptake. It would have been great if the author could also demonstrate the gaps at district level. Include the prevalence, retention rate, and EID uptake for the study districts in the introduction section. These will help justify why the two districts were included in the priority districts for HIV/AIDS interventions.* | Thank you for this comment. We agree and have added some information in the Introduction on retention and EID uptake, as well as clarifying that these are intervention districts for EGPAF. |
| 1 | *The Methods section did not clearly state the significance level of the study error margin (e.g. 0.05 as significance level).* | This is useful feedback. The p-value was (p<.05) was added to the Statistical Analysis section. |
| 1 | *The author may also wish to be more systematic : define both the primary and secondary outcomes ; then univariate analysis ; bivariate analysis and then multivariate.* | Thank you for this suggestion. We feel the way the results are currently presented is the most straightforward, so we are maintaining this section as is. |
| 1 | *In terms of the study broader objective, the author ought to be consistent throughout the document: to use assess rather that describe or evaluate.– the author has used the term ‘describe ’ and ‘Evaluate’, but it seems the author means to say ‘assess’ (on p. 6, line 74 and line 104).* | Thank you for this useful suggestion. We have changed the instances of “describe” and “evaluate” to “assess.” |
| 1 | *The actual implementation of the intervention was summarized in such a way that it is difficult for the reviewer to understand what actually happened on the ground. Additional information on actual implementation of CSC is required.* | Thank you for this suggestion. We have added a few more clarifying details in the Intervention Description. We have also referenced the paper that was written on the adaptation that provides more details. |
| 1 | *The variables tracked in the score card are not specified, this includes the indicators of issues that were identified and also the actual scores during the baseline and thereafter. Inclusion of score card indicators could have set a good background of the evaluation.* | Thank you, we agreed with this suggestion and added a few examples of issues identified to the Intervention Description. |
| 1 | *The paper should clearly indicate the data management system that was introduced during the actual implementation of CSC and the evaluation could have also identified the gaps and recommend proper data management system for CSC rather than included that as an area of research.* | Thank you for flagging that this was not clear. We have added some information at the start of the study design description to clarify that we used existing clinical records. |
| 1 | *One sentence in the Data section must be revised to be grammatically correct(P.11; Line 184)* | Thank you for the careful read. However, we did not find any grammatical inaccuracies in this section upon review. |
| 1 | *It is not clear how informed consent for the minors (15-17 years) was done* | Thank you for noting this. We added this sentence in the Ethical Approval section: In Malawi, pregnant women aged 15-17 years are emancipated minors and thus can provide informed consent for themselves and their infants. |
| 2 | *The data visuals are not overly appealing for an audience. Consider revising Table 2 into a graphic to show the pre and post results. Even a non-significant finding will still capture the audience’s eye faster in a graphic than a table. (not required, just for consideration)* | Thank you for your suggestion. However, we believe that the table efficiently sums up and presents the necessary information |
| 2 | *Line 144-145. This sentence was unclear to me. "A subset of women who participated in the survey were randomly approached using a number, n, 2-6 for a FGD that took place at a different date and venue." Please explain what is meant that the women were approached with a number. Perhaps they were randomly assigned a number?* | Thank you for noting this. We have clarified this sentence to indicate that the women were randomly assigned a number. |
| 2 | *Line 154. Should "consenting" be "consent"* | Thank you for this edit. We changed this to consent. |
| 2 | *Line 249: the authors suggest that the reviews of the coded transcripts were done throughout the process but then mention three distinct time points. It seems like the sentence should either say “throughout the process” or at the three time points, but not both. Also you could consider providing a sentence on what was done to ensure inter-rater reliability or at a minimum how many different team members were doing the coding.* | Thank you for this suggestion. We have edited the Qualitative Analysis section to provide additional details on how the coding was conducted. |
| 2 | *Line 291: it was interesting how different retention results were for women already on ART vs women starting ART in this study vs the previous research noted in the start of the document. Please comment on that in the discussion.* | Thank you for the interest. We have commented on this in the Discussion. The previous research compared retention of PMTCT women with non-pregnant women initiating ART. We did not make this comparison in our study. |
| 2 | *Lines 459-464. Should this concluding statement come after the limitations – so explaining what was found despite the limitations mentioned before?* | Thank you for this suggestion. We have added a Conclusions section following the limitations and have added this text to the Conclusions. |
| 2 | *To meet the data transparency and availability requirements, I believe the statement that data are available from EGPAF should be included within the text of the manuscript.* | Thank you for noting our omission. We have added a statement on data transparency. |
| 3 | *The title of the paper could benefit from including what the outcome is so readers know from the onset what is being measured and it also sets the stage for what is follow in the paper – what is the intention of the score card and what are you expecting to change among your target population?* | Thank you for this suggestion. We felt that simplicity in the title was preferred. |
| 3 | *Still on the title, the study was done in a couple of districts and not the entire Malawi, the authors can be more precise by naming the districts or simply saying “in two districts in Malawi”.* | Thank you for this suggestion. We did add “in two districts in Malawi” to clarify the scope of the intervention. |
| 3 | *The abstract and body of the paper could benefit from inclusion of participant selection or sampling method and criteria* | Thank you for this suggestion. We have added more sampling and inclusion information to the abstract. We have more clearly indicated the sampling method for women participating in the surveys and FGDs in the Methods section. |
| 3 | *Were the facilities compared all at the same level and what is the implication of lumping together different level of care in one analysis – any risk for of bias? Better description on the distribution of sample by level care (if any) may also help understand any nuances that may be confounders to adjust for.* | Thank you for this question. We adjusted for the different types of facilities, (hospital/health centers) in the analysis, through the logistic regression, and have specified that in the Statistical Analysis section. |
| 3 | *Did you have a same or separate sample pre and post intervention?* | Thank you for this question. This was not a cohort study, so have tried to clarify that the pre and post were based on time period. |
| 3 | *Still on the intervention I wasn’t sure if you used the same cohort for the different rounds of the CSC and if that has implication for the outcome of the study?* | Thank you for this question. We added language to Intervention Description to clarify that the participation in the CSC varied. We also added information to the Methods to further clarify that all women were eligible for abstraction, even if not participating in the CSC. |
| 3 | *I do not think that CSC is appropriate for some of the outcomes given what CSC is intended for and what it can not do. In this regard, the background could benefit from a richer description of what the impediments to PMTCT service outcome are and which of them are amenable to CSC and make the study about such outcome. It is unsurprising that it had no impact on retention or EID given that their known determinants are more than relationship and power interactions and also includes psychosocial and structural factors beyond the competence and scope of the health care workers. The lower rate of retention of newly diagnosed HIV clients is one example to show that what affects retention is beyond service delivery and access.* | Thank you for this critique. We chose to test the CSC in a PMTCT setting as it has already been used for other health care issues and has been shown to be an effective approach for improving provider-client relationships, and thus perceived and actual quality of care. We do agree that this was not as clearly spelled out in this paper so we have added more information to the Introduction to clarify our use of the CSC, indicating that provider-client relationships can be a barrier to PMTCT and that CSC is a tool that can be used to improve these relationships, and thus clinical outcomes. |
| 3 | *With the above I am uncomfortable with the mismatch of problem and CSC solution. I feel the paper can be better structured from start to finish focusing on what CSC can achieve given the preponderance of evidence of what drives various PMTCT outcomes.* | Again, thank you for flagging this. We have not changed the structure of the paper, as there is an accompanying paper that provides more information on the use of the CSC in this situation. However, we have added more information to the Introduction, as previously noted. |
| 3 | *The six aims of improvement suggested by the Institute of Medicine may help to position element of improvement CSC can address and should be focus of the paper, otherwise will be an unfair or an insufficiently considered assessment – see link for application of IOM’s six aims of improvement* [*https://doi.org/10.1097/01.pcc.0000160592.87113.c6*](https://doi.org/10.1097/01.pcc.0000160592.87113.c6)*.* | Thank you for this resource. The accompanying CSC paper referenced uses the Kruk et al high quality health system framework, which is similar to IOM. We were testing whether this process could affect joint actions to address poor service quality. It is also well-suited to address patient-centeredness. |
| 3 | *In terms of the FGDs, will post intervention FGD of retained client not be biased to the views of those who were retained and may be exaggerate the intervention’s benefits?* | Thank you for this question. We agree this is a potential limitation, and we have added the text to the Limitations section. |
| 3 | *Who conducted the data abstraction and interviews?* | Thank you for this request for clarification. We have added more text to make it clearer that the data collection activities were conducted by research assistants. |
| 3 | *How was confidentiality maintained in both instances particularly the FGD of people living with HIV?* | Thank you for this suggestion. We agree and have added some information in Methods on how confidentiality was protected. |
| 3 | *Why did you need ethics wavier for data abstraction if you had access to participants for your intervention why did they not consent to your using their data?* | Thank you for this note. We added some language to clarify why consent was not sought in the Ethical Approval section. |
| 3 | *Who conducted the intervention?* | Thank you for this question. We have clarified in the Intervention Description that the CSC was implemented by CARE-Malawi staff. |
| 3 | *What kind of actions were implemented to address issues emerging from the CSC?* | Thank you for this suggestion. We agree and have added some examples to the Intervention Description. |
| 3 | *How were the actions tracked to see if they changed before assessing impact? If nothing changed, can it be attributed to the CSC or other elements?* | Thank you for this suggestion. We have included some information in the intervention description, and the existing qualitative results note perceived improvements. |
| 3 | *It may help to have a table of outcomes, data collection tools and reliability of the different scales. It is hard to follow from the narrative.* | Thank you for this idea. We have included the scales used and their reliability in a table, replacing the text. |
| 3 | *The description of qualitative analysis method could be improved to demonstrate process and stages of analysis, by who, quality assurance and trustworthiness elements etc.* | Thank you for this suggestion. We have added more details about how the analysis was conducted in the Qualitative Analysis section. |
| 3 | *The mixed methods used approach should be specified given its implication for data analysis and presentation, I may be wrong but it appears to me as a concurrent triangulation and would have preferred to see a greater mixing or integration of both quantitative and qualitative data rather than the observed clear separation* | Thank you for this critique, and we appreciate this idea. In the design, these were unique components that were then brought together at the analysis phase to provide context and clarity. |
| 3 | *Given what know about PMTCT today this paper may be more novel if it focuses on its elements of collective efficacy and with better description of the intervention, study design and methodology.* | Thank you for this comment. Collective efficacy was one outcome of interest, but is not the only outcome associated with the CSC. We felt that the more novel aspect was a holistic evaluation of the intervention. Fewer studies in the literature have tried to link community action and clinical outcomes, which is one of the aspects we were assessing. As such, we have chosen to retain the focus of this paper as providing a broader view of the CSC’s performance. |
