# Supplementary material for: Evaluating the effect of a community score card among pregnant and breastfeeding women living with HIV in two districts in Malawi
Source: PLoS One. 2021 Aug 11;16(8):e0255788. doi: 10.1371/journal.pone.0255788 (PMC8357126; doi:10.1371/journal.pone.0255788)
Supplement: Attachment — Submitted filename: Malawi CSC response to reviewers 7.21.21.docx [file pone.0255788.s010.docx]

| Reviewer | Comment | Authors’ Response |
| --- | --- | --- |
| 4 | The abstract could be improved by including the figure for low retention rate, and care continuum for PMTCT in Malawi. This may provide a stronger rationale for researching the complimentary CSC intervention. | Thank you for this comment. We omitted this due to word limits in the abstract. Unfortunately, there is insufficient space to include this. |
| 4 | Study design and selection of Health facilities and hospitals needs to be provided in the methods section of the abstract. How were the districts and facilities sampled? Stratified random sampling based on public/private facilities? Both district hospitals? | Thank you for this comment. Given the limitations on the length of the abstract we have limited space to describe this but have clarified that the health facilities were purposively selected in the abstract and provided additional detail in the Study Setting section of the Methods. |
| 4 | The key limitation of intervention duration may be mentioned in the conclusion. | We added a note on the relatively short intervention period in the final paragraph of the abstract and retained our more comprehensive reflections on this in the Discussion section. |
| 4 | Women did not have to participate in the CSC intervention to be included in the abstraction, as it was hypothesized that CSC-related clinical improvements may affect the quality of care, PMTCT retention, and EID uptake for all PMTCT clients” This sentence illustrating the selection of the sample, seems to be contrary to the principles of the CSC where engagement, ownership to the process leads to change, uptake of services or perceived quality due to visible improvements as illustrated by the scorecard. | Thank you for this comment. While we certainly think many outcomes of the CSC are likely most strongly experienced by those who participate directly in the process, such as increased self and collective efficacy, we hypothesized that improvements in quality of services brought about by the CSC *might* be experienced by all clients and then lead to improvements in retention and uptake. As such we analyzed this using an intent-to-treat approach—among anyone who could have participated in the CSC, even if they decided not to. We did specifically target those participating in CSC through FGDs and surveys to measure engagement and perceived quality, as noted in the sample section. |
| 4 | The authors may consider including a CSC model and describe the details of the process. References are provided but further explanation on the implementation of the generic model in the Malawian context is needed. How was the CSC introduced, were facilitated meetings scheduled for joint discussion and deliberation of results and the rationale for scoring poor, or high. | Thank you for this comment. Because the CSC model is described at length in other referenced, open-source materials, we have decided not to repeat a detailed description but at the reviewer’s suggestion we have included a Figure that illustrates the process including the coming together of stakeholders in the interface meeting phase for joint discussion and deliberation as the author describes. |
|  | Experience of HCW was as low as 1m? HCW has minimal time to engage with communities, due to the workload, shortages in staffing, and high priority services. It is unlikely that HCW who have 1m experience will be able to effectively address community needs, or challenges with the PMTCT demand side challenges without prior experience. This needs to be discussed as a limitation. | Thank you for this comment. The one month mentioned here refers to the minimum amount of time an eligible health worker could be working at a study facility not working in the field of health care more broadly. The majority had been in their position for 2+ years. A sentence to this effect has been included in the text. |
| 4 | Who were “other” HCW cadre? | We have included this information in a footnote below the table. |
| 4 | CARE has a well established CSC model that has been successfully adapted in other contexts. However, the period of intervention (1y?), maybe insufficient to establish the various community mechanisms and systems at the facility level to ensure optimal operations and internalization of the concept and procedures. | Thank you for this comment. We agree and have updated the language in our Discussion section to the following: “In actual practice, the CSC process typically includes multiple rounds with longer action periods between rounds over the course of multiple years. These longer implementation and action periods and additional rounds may be needed for clients, HCWs and governments to effectively mobilize and implement transformative solutions, particularly operational mechanisms and procedures at the facility-level, and for these to translate into changes in behaviors and practices.” |
| 4 | The CSC methodology needs more detailed description to improve the study rigor. The discussion or methodology does not include measures for implementation integrity and intensity of the CSC. Inclusive representation in the community CSC. | Thank you for this comment. As noted above, the CSC methodology for this study is described at length in an accompanying paper. We have added the figure to provide additional clarification on the approach, but for further details on the specifics of the implementation, we would like to refer readers to that paper. We have added further language to the limitations: “There was also variability in CSC implementation by site, which may have affected the outcomes. Variations in implementation and CSC actions by site were captured but were not included as a factor in the analysis as the study was not powered to analyze results by site.” |
| 4 | The facility aspects need to be optimized and integrated, as ART and maternity information was not available in one HF. | We agree that information availability is an issue for clinical data in Malawi, and efforts are underway to address this. |
|  | Level of engagement and contribution in meetings needs to be measured, rather than generic indicators for meeting participation. | Thank you for this comment. We agree that this information is of interest, but observation was not a component of this evaluation. Rather, we used the focus group discussion to elicit their impressions of the CSC process. |
| 4 | Were the 606 women assessed in the post evaluation included in the 627 reported in the pre evaluation | Thank you for this question. In the inclusion criteria, it specifies: “For the abstraction of data from existing clinical records, women were eligible for inclusion if they were HIV-positive (newly or previously diagnosed), aged 15 years or older, and attended their first ANC appointment and received ART between August 2016-March 2017 (pre-intervention) and between August 2017-March 2018 (post-intervention) in one of the 11 selected facilities. All eligible women were identified using the clinic’s ANC register and assigned a unique study identifier.” As such, each is a point in time measurement. This is not a cohort study. We have included the word “cross-sectional” in the design section to further emphasize this. |
| 4 | 75% had data available in pre and 93-94% in post, was this improvement a result of the CSC? | No, this was not a focus of the CSC. This is a result of ongoing support to clinics to improve clinical recordkeeping. A sentence on this was added to the limitations. |
| 4 | How did the HCW participate in the CSC, what was their specific role and continuing responsibility, were HCW designated to facilitate follow up with the community? Were problem solving mechanisms or action plans instituted following the CSC mtgs? Was there an open community forum to discuss and deliberate results? | HCWs participated in the CSC as service providers. We believe this is more clear with the addition of Figure 1 (see comment above). HCWs identified priority issues in the delivery of PMTCT care, scored these issues as qualitative indicators, presented these scores in a joint interface meeting with service users (PMTCT clients) and other stakeholders, and were part of the process to develop, monitor and implement a joint action plan. |
| 4 | The authors have embarked on a critical people centered paradigm for enhancing health improvements using the CSC model. However, the mechanisms of implementation integrating an inclusive approach of patients and HCW need careful consideration and deliberation, for a meaningful interpretation of the effectiveness for health outcomes and health system improvements. Patient perception, satisfaction, perceived reduced discrimination must be weighted equally with the validated outputs/outcomes for the PMTCT utilization, continuum of care and quality, for a value based healthcare system. Harsh, disrespectful, unpleasant provider patient encounters need to be valued and integrated in quality of care framework. | Thank you for this comment. This is an excellent point and we explored these aspects of patient-centered quality of care, which did improve significantly in another paper that presents this work. We have revised our conclusion to emphasize this point. It now reads as follows:  “Social accountability approaches like the CSC have demonstrated that they are well-positioned to impact important patient-centered elements of service quality such as patient perception, satisfaction, and trust and communication between client and provider, that are often under-valued and neglected in quality-of-care frameworks. These elements are often within the direct control of individual HCWs, facilities, and, to some extent, community members, and so may be more amenable to change through a process like the CSC, particularly when implemented over just a short period of time. While these valuable contributes of these approaches should not be dismissed, especially where efforts to improve patient-centered dynamics of care have fallen short, they may not be the ideal mechanisms to address systemic and interpersonal issues that are often critical to PMTCT retention and EID uptake, particularly in a context of stigma or other individual-level barriers.” |
| 5 | Were the clients who were recruited utilizing services from the selected facilities? Where were these clinics and associations of people living with HIV selected from? | We have modified the language to clarify that the clinics are the intervention clinics and the associations are from the catchment areas. |
| 5 | Did the HCWs and PMTCT clients identify the issues and barriers most impacting their ability to successfully deliver and access quality of PMTCT services jointly or did HCWs do it alone and clients identified theirs alone? That part is not clear in the write-up | We have added a figure (Figure 1) that provides greater clarity on the CSC process and the roles of each. |
| 5 | What was the aim of revisiting previous issues? | These were revisited in order to assess progress made as a result of the action period that proceeded it. We have clarified this in the description. |
| 5 | Was it only community leaders or community leaders and community members? | Thank you for this question. Because this CSC was focused quite specifically on PMTCT services it was just community leaders, those who are most in the position to commit to and implement solutions / action items. Accordingly, no changes were made to the text. |
| 5 | I would think that for one facility, the quota should be 12 and not six as stated. Please clarify on this | Thank you for this request. Please see updated language in the methods section. We have removed discussion of the quota system to explain how participants were selected and how many facilities FGDs were conducted at. We think this language is clearer for the reader. |
| 5 | From what? | Thank you for this question. From the registers. We have made a small adjustment to the language to reinforce this. |
| 5 | Was ANC the point of identification of the clients who were included in the study? | Thank you for this question. The answer is yes, and this is described in the study Study Participants and Sample Size section: “All eligible women were identified using the clinic’s ANC register and assigned a unique study identifier” |
| 5 | This sounds like a result which should be in the results section. | Thank you for this suggestion. We agree and have moved it to the clinical abstraction results. |
| 5 | How do define social cohesion? | Because this is not a result we report on in the paper, we did not include the scale (see below), but I have added the short definition in parantheses.  FYI: Reference 26, CARE USA. (2014) Health Workers’ Voices in Open, Inclusive Communities and Effective Spaces (Health Worker VOICES): *A tool to measure governance outcomes in sexual, reproductive and maternal and child health programs.* Atlanta GA, defines social cohesion with a scale which measures perceptions of mutual aid, trust, connectedness and social support.”  There are 11 items in this scale: 1. I cannot rely on the people I work with to give me advice.* 2. I can rely on the people I work with to help me out with a difficult patient. 3. Oftentimes there is conflict among the people I work with.*   4. I enjoy the people I work with. 5. In general, the people I work with only worry about themselves.* 6. I can rely on the people I work with to stand up for me if I point out a problem at work. 7. I can rely on the people I work with to support me when I try to improve my performance at work. 8. I can trust the majority of people I work with 9. In general, the people I work with get along well. 10. The people I work with will resent me if I try to do things to improve the quality of care in this area/facility.* 11. The people I work with treat me with respect. (*Reverse scored). |
| 5 | How many research assistants facilitated the FGDs for PMTCT clients and how many research assistants facilitated FGDs for HCWs? | There were seven research assistants that facilitated the FGDs across both groups. This information has been added. |
| 5 | What were the scores and How were the scores obtained. That is not explained Results | Thank you for this question. As the word “score” may have been creating confusion, we have updated this to say “values” or “scale average.” The explanation of the calculation is in the measures section (averaging the values of the scale items). |
| 5 | Mean ages should be reported with corresponding standard deviations | Thank you for this comment. Previous reviewers asked us to present the ages in age categories rather than means, and we have thus removed the means from the text. |
| 5 | The table should include significant results | Thank you for this comment. We ultimately decided this was not a meaningful measure despite being significant, as it measured participation in community activities, which is equivalent to the CSC. Thus, we are not highlighting it in the table. |
| 5 | Was it a requirement for these participants not to share their socio demographic characteristics? | Thank you for this question. It was not a requirement, which is why we noted that six chose not to. |
| 5 | It’s appropriate to report qualitative data in themes and sub themes and then you compare those themes pre- and post-intervention. This could be based on the different aspects that authors set out to evaluate e.g various aspects of PMTCT services.  It would be appropriate to first report the relationship that existed previously before the intervention was implemented and then the authors can report improved relationship after the project has been implemented. | Thank you for this comment. As this is a mixed methods paper, with the qualitative component being just one part, we decided to summarize both sets of data (pre and post) and provide examples from both sets of data. The authors feel this is the best way to present the information succinctly in complement to the quantitative findings. |
| 5 | Is it possible to have a quote for this? | Thank you for the suggestion. We have added a quote. |
| 5 | Who mentioned this; was it the HCW or the participant? Also note that at the end of each quote you should specify who said that quote. | Thank you for the question. As noted at the beginning of sentence, this sentiment was shared by a HCW. The information regarding who said the quote is provided before the quote, to introduce it. |
| 5 | Is it possible to get a quote indicating that the HCWs felts that the clients did not appreciate their work? | Thank you for the suggestion. We have added a quote. |
| 5 | Add whoever mentioned this quote at the end of the quote | Thank you for this comment. This information is provided for each quote, but it is provided immediately before the quote to introduce it, as opposed to after it. |
| 5 | Where is the quote to sow that the HCWs reported a greater understanding of the clients’ feelings and their needs? | Thank you for the suggestion. We have added a quote. |
| 5 | Lines 406-7; 415-18: Same comment as above. | We are not sure what the reviewer intends here, as the lines indicated are currently a quote. |
| 5 | Line 419-23-Is it possible for the authors to back this statement up with a quote | Thank you for this suggestion. There were two quotes mentioned in this section, but they were not italicized, so it was not clear that they were quotes. We have now italicized them. |
| 5 | Line 424-7-Could we get a quote from the HCWs to reflect this and also one from the PMTCT clients | Thank you for this suggestion. Quotes are used to provide a participant voice when needed. This finding identifies concrete actions that occurred, so the authors feel that personal voice is not needed. |
| 5 | What does corrected information about HIV and ART mean? | Thank you for catching this error. This was a typo and should have been ‘correct.’ This edit has now been made. |
| 5 | Line 435-37-Include the participant who mentioned this quote at the end of the quote | This information is provided for each quote, but it is provided immediately before the quote to introduce it, as opposed to after it |
| 5 | General comment for the Results section: it’s usually appropriate to report results concurrently where mixed methods is used. Could the authors explore that possibility? | Thank you for the suggestion, but we feel that the results are presented more clearly when presented separately, especially given the disparate findings. |
| 5 | This is what the authors set out to find out before the intervention was implemented. So after the intervention what did the authors find out?  Also the authors should be able to make the introductory section in the discussion section in line with the objectives of the study reporting significant findings in the first paragraph of the discussion section before going ahead to discuss each of the results in detail in the later paragraphs of this section | Thank you for this comment. The objectives and findings were explained in the first two paragraphs, but this has been revised to include a first paragraph with the objectives and results briefly summarized. |
| 5 | This was the finding of the study but what does it imply? It would also be appropriate to give other studies with similar or dissimilar results when you are discussing your findings. | Thank you for this suggestion. We have provided additional references, and the existing discussion includes comparisons to other studies. |
| 5 | Line 459-460: The authors should provide a reference for this statement. | Thank you for this comment. This is the hypothesis/explanation of the authors related to this specific study, as is appropriate to the discussion, so there is no reference to include. |
| 5 | What brought about this? Did it have any effect on the key findings of the study, if so then explain, giving other studies with the same or different findings | Thank you for the question. As literature suggested different rates of retention by new vs. previously diagnosed, we analyzed these separately. The intervention did not improve retention for either group. Text has been added to the discussion clarifying that. |
| 5 | Is 318 a reference or not? If it’s a reference, please correct it because reference 318 does not exist in the reference section. | Thank you for catching this typo. This is reference 31. We have corrected this. |
| 5 | Long sentence. Consider fragmenting it | Thank you for this suggestion. We have edited this section. |
| 5 | Also what makes the authors think that CSC is not an ideal mechanism to address systemic and interpersonal issues? Are there studies that suggest this? Please clarify and give a reference | Thank you for this question. This is the author’s hypothesis and builds upon the previously cited work showing lack of changes in clinical indicators using CSC or other community processes. We have not edited as we do not want to re-cite the same references included earlier in the paragraph. |
